# Wearable Insulin Biosensors for Diabetes Management: Advances and Challenges

**DOI:** 10.3390/bios13070719

**Published:** 2023-07-07

**Authors:** Sotiria D. Psoma, Chryso Kanthou

**Affiliations:** 1 School of Engineering & Innovation, The Open University, Milton Keynes MK7 6AA, UK; 2 Faculty of Medicine, Dentistry and Health, University of Sheffield, Sheffield S10 2RX, UK; c.kanthou@sheffield.ac.uk

**Keywords:** insulin, biosensor, diabetes mellitus, continuous monitoring, wearable biosensor, immunoassay, optical, electrochemical, aptamer, molecularly imprinted polymer (MIP)

## Abstract

We present a critical review of the current progress in wearable insulin biosensors. For over 40 years, glucose biosensors have been used for diabetes management. Measurement of blood glucose is an indirect method for calculating the insulin administration dosage, which is critical for insulin-dependent diabetic patients. Research and development efforts aiming towards continuous-insulin-monitoring biosensors in combination with existing glucose biosensors are expected to offer a more accurate estimation of insulin sensitivity, regulate insulin dosage and facilitate progress towards development of a reliable artificial pancreas, as an ultimate goal in diabetes management and personalised medicine. Conventional laboratory analytical techniques for insulin detection are expensive and time-consuming and lack a real-time monitoring capability. On the other hand, biosensors offer point-of-care testing, continuous monitoring, miniaturisation, high specificity and sensitivity, rapid response time, ease of use and low costs. Current research, future developments and challenges in insulin biosensor technology are reviewed and assessed. Different insulin biosensor categories such as aptamer-based, molecularly imprinted polymer (MIP)-based, label-free and other types are presented among the latest developments in the field. This multidisciplinary field requires engagement between scientists, engineers, clinicians and industry for addressing the challenges for a commercial, reliable, real-time-monitoring wearable insulin biosensor.

## 1. Introduction

A century ago, the Nobel Prize in Physiology or Medicine was awarded jointly to Frederick Grant Banting and John James Rickard Macleod for the discovery of insulin and its relationship with diabetes [1]. Even though this discovery has saved millions of lives, according to the International Diabetes Federation (IDF), one person dies of diabetes every 5 s worldwide [2]. IDF statistics for 2021 show that 537 million adults (20–79 years) are living with diabetes, nearly 1 in 10, and this number is predicted to rise to 643 million by 2030 and 783 million by 2045 [3]. Diabetes mellitus is a chronic metabolic disorder characterised by the inability of the β-cells of the pancreas to produce enough insulin [4]. In Type I diabetes, the pancreas is incapable of producing insulin, while in Type II diabetes, the pancreas does not produce enough insulin and/or the body becomes resistant to it. Type II diabetes is treated with insulin while Type II diabetes can be treated with drugs that lower glucose independently of insulin. Eventually, many Type II diabetic patients also become reliant on exogenous insulin for regulating their glucose levels. 

Diabetes management requires the regular checking of blood glucose levels and, for more than four decades, glucose biosensors have been in use by diabetic patients [5,6,7,8]. However, measuring glucose is an indirect method for calculating the required insulin dosage and failure to administer the correct amount of insulin can lead to severe hypoglycaemia or hyperglycaemia with serious consequences to health. Continuous insulin and glucose monitoring would enable a more accurate estimation of insulin sensitivity, regulate insulin dosage and facilitate progress towards the development of a reliable artificial pancreas [9,10,11,12,13]. Although several insulin detection devices have been developed, many challenges remain, associated with establishing specificity, enabling detection at the nanomolar range and avoiding interference from endogenous molecules. At present, the needs for miniaturised, low-cost, easy-to-use and reliable insulin biosensor platforms remain largely unmet. Several methods suitable for point-of-care insulin detection, mainly direct electrochemical and optical methods such as fluorescence, are reviewed. The challenges and limitations regarding specificity, sensitivity and appropriate detection limits of human insulin are explored. Nucleic acids such as aptamers, molecularly imprinted polymers and nanomaterials like modified carbon nanotubes [14] that have high affinity and specificity towards insulin offer promising exciting possibilities for enhancing sensitivity, lowering detection limits and improving insulin biosensor stability. 

Here, we review current research and future developments and challenges. Ultimately, point-of-care simultaneous real-time measurements of insulin and blood glucose using biosensor technology can offer significant potential for minimising the impact of the impending diabetes epidemic and improve the quality of life of diabetic patients.

## 2. Insulin Structure and Function and Its Detection in Biological Fluids

Insulin is a 5808 Da peptide hormone produced and secreted by the pancreas in response to increased levels of glucose in the circulation. It consists of a 21-amino-acid A chain and a 30-amino-acid B chain held together by two disulfide bonds and is responsible for regulating carbohydrate, lipid and protein metabolism by stimulating the uptake of glucose through insulin receptors found mainly in peripheral muscle, in adipocytes and in hepatocytes. Impaired insulin production and secretion and/or a reduced response to insulin, also known as insulin resistance, are key underlying mechanisms leading to the development of type II diabetes [15,16]. To compensate for the resistance to insulin, the pancreas secretes more insulin into the circulation (hyperinsulinaemia). Although moderate levels of insulin resistance can be beneficial for ensuring the supply of glucose to the brain [17], exacerbated levels of resistance can result in chronic hyperglycaemia, leading to prediabetes and type II diabetes. Insulin resistance can be evident long before the development of diabetes and thus elevated fasting levels of insulin can potentially predict the onset of this metabolic disorder [18]. 

Insulin can be measured in blood serum and plasma, but it can also be detected in other biological fluids such as saliva, tears or sweat [19,20,21]. Measurement of insulin is, however, challenging mainly because it is present at very low concentrations in serum and plasma, and this requires very sensitive techniques (Table 1). At normal fasting conditions, the insulin concentration in serum can be below 50 pmol/L while concentrations of >70 pmol/L indicate insulin resistance and the onset of type II diabetes [22,23]. In interstitial fluid, insulin concentrations are on the order of 20–50% lower than those in plasma [24,25,26]. Similarly in saliva, insulin levels were shown to be consistently lower than those of plasma [20,27,28]. Fabre et al. [20] studied saliva in children where insulin levels were as low as 10% of those found in plasma. Messenger et al. [28] reported even lower salivary insulin levels than plasma close to 50%. 

## 3. Difficulties and Limitations of Current Insulin Management Techniques

Blood glucose levels in healthy non-diabetic individuals range between 3.9 mmol/L (70 mg/dL) and 5.6 mmol/L (100 mg/dL) after fasting and rise up to 7.8 mmol/L (140 mg/dL) two hours after eating (World Health Organization data). The pancreas secretes insulin at constant low levels to keep glucose steady and responds by increasing insulin secretion when blood glucose rises after a meal. The finite regulation of insulin secretion is disrupted in diabetes, which is diagnosed when fasting glucose levels reach 7.0 mmol/L (125 mg/dL) or above. Chronic hyperglycaemia can cause microvascular damage and contributes to various serious pathological conditions including retinopathy, nephropathy, neuropathy and cardiovascular disease [33], while severe hypoglycaemia can be life-threatening. It is therefore important to maintain blood glucose within a target range. Exogenous insulin aims to mimic the physiological secretion of the hormone, but there are challenges associated with administering it at appropriate levels to achieve this while avoiding episodes of severe hypoglycaemia [34]. 

Calculating the insulin administration dose is based on intermittent blood glucose measurements, an estimate of the amount of carbohydrate consumed and on predictions of how endogenous and exogenous insulin would respond. Various synthetic insulin analogues have been designed with distinct pharmacokinetic and pharmacodynamic properties including long- and short-acting analogues, and their use has led to some improvements in glycaemic control of diabetic patients [35,36]. Further advancements in continuous glucose monitoring facilitated by the development of wearable biosensor devices have enabled more effective management of diabetes [37,38]. However, although biosensors enable glucose monitoring in real time, estimations of insulin dosage are still indirect [39]. Biosensor devices measure glucose in interstitial fluids rather than blood and glucose values can vary between the two compartments especially during periods of physical exercise or after a meal [40]. An individual’s weight and their sensitivity to insulin as well as other factors such as hepatic and renal function or any medication they are on can influence how much insulin is needed to ensure glycaemic control. The most common route for insulin administration is via subcutaneous injection using a vial and syringe or an insulin pen. 

Glucose-responsive insulin delivery systems represent recent advancements in this field and are based on continuous glucose monitoring that incorporates a closed-loop system delivering insulin via a pump [41,42]. While these systems are a step forward in achieving better glycaemic control, similar challenges to those described above apply and closed-loop systems are still not fully automated, since the user needs to input information about their carbohydrate intake. There is therefore a clear need to develop wearable devices, similar to glucose monitors, to detect insulin in real time that can be incorporated into a closed-loop system.

## 4. Conventional Insulin Detection Methods

Immunoassays and chromatography are the two main selective and sensitive conventional methods used nowadays for the detection of insulin in blood, with immunoassays being the most commonly used in the clinical setting [43]. 

The Enzyme-Linked Immunosorbent Assay (ELISA) is a commonly used immunoassay for insulin detection in the clinic [10,44]. It employs capture and detection antibodies, each recognising a different epitope on insulin with the detection antibody coupled to a reporter enzyme system that yields a quantifiable signal, usually detected spectrophotometrically. Chemiluminescence immunoassay (CLIA) methods are also commonly used for insulin detection and utilise a similar principle to that of an ELISA except that detection is through a chemical reaction that generates light [10,45]. Many commercial immunoassay kits are now available for quantifying insulin and some employ sophisticated automated systems for sample processing through to data processing [46]. Immunoassays are high-throughput and relatively cost-effective but are time-consuming and lack sufficient standardisation and compatibility across different analytical procedures [46]. 

Chromatography-based methods are used in the clinical detection of insulin and have the advantage of distinguishing between insulin and its various degradation products and different insulin analogues that are used in the management of diabetes [43]. HPLC separation combined with UV detection or spectroscopy-based methods is one of the most widely tested analytical methods for insulin detection [43,47]. Advantages over immunoassays include significantly faster detection times and improved selectivity and sensitivity although sample preparation is elaborate and bulky equipment is required. The main difficulty though with the aforementioned classic methods for insulin detection is that they are expensive to run, time-consuming and are not suitable for when immediate action to correct glucose or insulin imbalance is needed.

## 5. Biosensor Technology

Biosensor technology is a multidisciplinary field where biology, engineering and nanotechnology promise solutions for healthcare challenges enabling personalised medicine for disease prognosis, diagnosis and drug delivery. It combines cutting-edge technology with integrated platforms of microfluidics necessary for analyte delivery and real-time monitoring, and bioelectronics for fast and automated signal processing and analysis to enable decision making [48]. Typical microfluidic platforms are formed of polymers such as polydimethylsiloxane (PDMS), silicon or glass, and integrated micropumps. The fast evolution of lab-on-a-chip (LOC) technology where microfluidics, different detection methods and nanomaterials are integrated offers capabilities for multi-analyte, reliable and cost-effective point-of-care diagnostic platforms/biosensors with enhanced sensitivity and lower detection limits compared to conventional methods [49]. In addition, recent advances in 3D-printing technologies offer a number of advantages in biosensor production such as higher resolution and low cost, and enable end-user customisation and prototyping [50]. Further miniaturisation of bioelectronics and their placement close or under the microfluidic channels where reactions take place can significantly contribute to overcoming issues of lag-time in detection by the biosensor, which is important for closed-automated-loop insulin delivery systems [51]. These developments also support the commercialisation pipeline of innovative wearable biosensors. 

The ability to use a point-of-care sensor to measure insulin concurrently with glucose would allow for a much better assessment of endogenous insulin activity, enabling real-time adjustments in insulin dosing to be made while minimising the likelihood of occurrence of extremes of hypoglycaemia or hyperglycaemia. A point-of-care biosensor would be tailored to the needs of an individual allowing for diet and exercise patterns to be incorporated and applied into their insulin dosing plan. In recent decades, affinity biosensors based on antibodies, nucleic acid aptamers and molecularly imprinted polymers (MIPs) have emerged as powerful and promising point-of-care diagnostic devices. Particularly, electrochemical and optical affinity biosensors have attracted attention [52]. In the literature, most of the insulin biosensors are based on aptamers and molecularly imprinted polymers (MIPs), which imitate or improve on antibody-based biosensors [53,54,55,56,57,58]. They offer portability, fast response, selectivity, specificity, high stability and reproducibility, low detection limits and low production costs.

### 5.1. Aptamer-Based Insulin Biosensors

Aptamers are single-stranded nucleic acids that can form secondary and tertiary structures, and like antibodies, they can recognise proteins specifically and with high affinity. However, unlike antibodies, they are small, heat- and pH-resistant, less expensive to produce and have the added advantage in that they do not interfere with endogenous insulin antibodies that diabetic patients who receive insulin often develop. Aptamers are isolated from nucleic acid libraries by a process known as SELEX (systemic evolution of ligands by exponential enrichment) by incubating the library with a target ligand molecule, and they can subsequently be produced synthetically. They have been explored for their potential use in biosensors for insulin in diabetes management with promising results [59,60,61,62,63,64,65,66,67,68,69]. 

**Figure 1:**
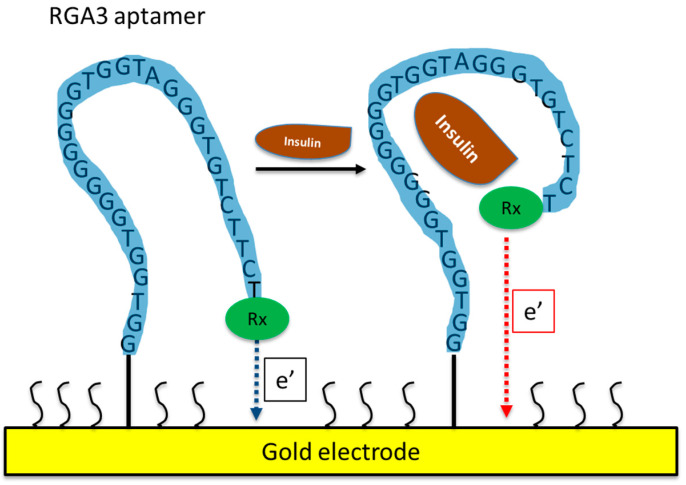
Schematic of an aptamer-based electrochemical insulin biosensor. Binding of insulin to the aptamer causes changes in its conformation and in electron transfer between a redox label and the gold electrode.

Using SELEX, Yoshida et al. [63] isolated several DNA aptamers against insulin (Table 2) and they selected IGA3 for further testing. IGA3 is a G-rich aptamer which folds into a G-quadruplex and demonstrates the highest affinity for insulin. An enzyme system using IGA3 coupled to a thrombin-inhibiting aptamer was designed so that upon binding of insulin, thrombin is released, and its activity can be measured in a clotting assay. Although enzyme activity correlated with insulin levels, the system lacked sensitivity and was not tested using physiological samples. Several studies have since used IGA3 as an insulin recognition element for further biosensor development [66,67,68,69,70,71]. Wu et al. [68] developed a more sensitive electrochemical insulin biosensor where IGA3 was immobilised on a gold electrode surface and binding of insulin resulted in a conformational change detected by changes in electron transfer. Insulin in buffer was detected to 20 nmol/L, with a rapid response time of 60 s. Furthermore, the sensor was specific and selective and therefore has the potential for further development for clinical applications. The basic principle of an aptamer-based electrochemical insulin biosensor is shown in a simplified schematic in Figure 1. Electrochemical insulin biosensors have also been developed based on the DNA sequence of the insulin-linked polymorphic region (ILPR), which is located in the promoter of the human insulin gene and therefore can be considered as a natural insulin aptamer, and low detection limits down to 50 nmol/L were achieved [63,66]. 

Sun et al. [67] developed a chemiluminescence biosensor for insulin also based on IGA3 and functionalised gold nanoparticles that are released upon the binding of insulin and catalyse the conversion of luminol. The biosensor was highly selective for insulin and had a low detection limit of 1.6 pmol/L. Wu et al. [69] found that insulin was bound to a C-rich-containing aptamer (cIGA3) with higher affinity and faster kinetics than the G-rich IGA3 aptamer, but specificity was poor for both aptamers and biological molecules such as haemoglobin and albumin were also bound. Their data contrast those by other investigators who found IGA3 to be highly specific for insulin with little interference by a range of molecules including albumin [67,68]. Aggregates of insulin can form and influence binding and, therefore, biological sample conditions need to be taken into consideration, especially when designing biosensors for clinical use [69]. Taghdisi et al. [65] developed a highly selective insulin biosensor utilising a triple helix switch consisting of another aptamer sequence that binds to insulin (IBA2, Table 2) and a fluorescent oligonucleotide probe. The probe is released upon insulin binding and results in the quenching of fluorescence. The sensor exhibited a low limit of detection of 9.7 nmol/L in serum and urine.

A comparison of detection techniques, sample applications, detection limits and linear concentration ranges of different aptamer-based biosensors for insulin can be found in Table 3. 

### 5.2. MIP-Based Insulin Biosensors

Molecular imprinting has received significant attention for nearly half a century. The fabrication of molecularly imprinted polymers (MIPs) involves the polymerisation of functional monomers around a template to form a cast with special recognition sites for the template. Upon removal of the template, the polymer can recognise the target molecule like antibodies recognise specific proteins. Molecular imprinting produces high-affinity polymers that can recognise not only proteins, but also nucleic acids, drugs and other targets and have several advantages over antibodies, as they are more stable, specific, easy and inexpensive to prepare and can be miniaturised for biosensor development.

Different molecular imprinting techniques can be employed in the construction of MIP-based sensors. These include bulk imprinting and surface imprinting [72]. In the first approach, the whole of the template molecule is imprinted in the polymer matrix and is then removed following polymerisation. While this approach increases selectivity for a specific molecule, in the case of insulin, smaller polypeptides and degradation products may also cross-react with the template. In addition, proteins may not remain in their correct conformation during the polymerisation process, thus also reducing selectivity. These drawbacks can be overcome by surface imprinting, where recognition sites on the surface of the template are used, are more accessible to the target and offer better binding kinetics. 

Using quartz crystal microbalances (QCMs) as biosensor transducers, Schirhagl et al. [73] compared insulin detection by natural antibodies with detection using either a directly imprinted surface polyurethane polymer or a double-imprinted insulin antibody replicate. The synthetic coatings showed insulin selectively like the antibody, with a detection limit below 1 μg/mL. However, the antibody replicate showed significantly enhanced sensitivity and can be produced in bulk in a cost-effective manner, making this technology attractive for further development. Kartal et al. [74] also developed a QCM-based sensor by imprinting a complex of N-methacryloyl-(l)-histidine methyl ester with insulin onto gold QCM chips. Detection studies were carried out using insulin in aqueous solution and in artificial plasma samples and demonstrated a linear relationship between 0.008 and 1 ng/mL, high selectivity and stability, and a low detection limit (0.008 ng/mL).

In 2020, Piletsky and his colleagues [75] synthesised insulin MIP nanoparticles and immobilised them on screen-printed platinum electrodes to form a stable insulin biosensor for clinical applications. The sensor was selective for insulin with a linear response over a range of 50 to 2000 pmol/L and a limit of detection of 81 fmol/L in human plasma. The sensor showed good stability at room temperature and can be mass-produced at relatively low cost, thus fulfilling many requirements for production of a point-of-care insulin sensor. Zidarič et al. [76] developed an MIP receptor using an electrochemical technique to polymerise pyrrol in the presence of insulin on a carbon electrode using cyclic voltammetry. They used single-drop analysis to detect insulin in pharmaceutical samples. The biosensor successfully detected insulin in linear concentration ranges from 20 to 70 pmol/L (R^2^ = 0.9991) with a limit of detection at 1.9 pmol/L. Wardani et al. [77] also developed an electrochemical insulin sensor with an even lower reported limit of detection of 33 fmol/L and a linear range of 0.05 to 1.4 pmol/L. They used a gold electrode modified with carboxylated multiwalled carbon nanotubes (f-MWCNTs) and MIP cryogel. The sensor was highly selective for insulin and was stable when stored at room temperature. Abstracted information of the main parameters on the above MIP-based biosensors for insulin detection is provided in Table 4.

### 5.3. Label-Free Insulin Biosensors

Another interesting category of insulin biosensors being developed is label-free and mainly employs electrochemical or optical transducers. Label-free detection is achieved through the intrinsic qualities of the target such as its charge, molecular mass and electrical impedance. Label-free systems offer simplicity at low costs and avoid a labelling stage that can result in sample degradation [78].

A label-free porous silicon-based optical biosensor was used to compare antibodies versus aptamers as bioreceptors for insulin, and interferometric reflectance spectroscopy (IRS) was used for detection [79]. Both antibodies and aptamers were highly selective for insulin although the aptamer-based approach demonstrated a faster response and lower limit of detection of 1.9 μg/mL. Servarayan et al. [80] presented a label-free fluorescence-based biosensor for the detection of insulin in human serum using novel naturally existing chromene mimic receptors. Its working range was from 10 fmol/L to 600 pmol/L, with a limit of detection of 7.07 fmol/L, which is reliable for the clinical detection of insulin. Chen and his colleagues [81] presented a label-free aptamer-based optical liquid-crystal (LC) biosensor for insulin. When an aptamer (IGA3) adsorbed to cetyltrimethylammonium bromide (CTAB) is bound to insulin, it undergoes a conformational change at the aqueous–liquid crystal interface, which results in a change in optical appearance from dark to bright that is then detected by polarised optical microscopy. This liquid crystal biosensor demonstrated a rapid response time of 5 min and high specificity and sensitivity for insulin in the range of 0.1–1.0 nmol/L in human urine and serum samples. Xu et al. [82] reported a label-free electrochemical sensor capable of ultrasensitive detection of insulin concentrations in blood serum. They immobilised insulin antibodies on gold electrodes and used electrochemical impedance spectroscopy (EIS) to monitor changes associated with the binding of insulin to the electrode surface. The sensor detected insulin across a clinically relevant range with a low detection limit of 4.7 pmol/L and was robust and could be regenerated without loss of sensitivity. Another EIS-based, rapid and label-free insulin biosensor with high sensitivity and accuracy was presented by Malcok et al. [83]. They used a similar principle to Xu et al. [57] of immobilising an insulin antibody to a gold electrode and used the imaginary impedance of EIS, to determine the optimal binding frequency (OBF) of insulin as 810.5 Hz and changes in imaginary impedance that correlated with insulin concentrations within a physiological range and with a low limit of insulin detection at 2.26 pmol/L. 

Gobi et al. [84] developed a surface plasmon resonance (SPR) immunosensor based on a novel surface functionalisation method of covalent immobilisation of insulin on a self-assembled polyethylene glycol (PEG) monolayer. They used the principle of a competitive immunoassay using an antibody to insulin and demonstrated high sensitivity, specificity in detecting insulin with a response time of less than 5 min and a low-detection-limit of 1 ng/mL. The immunoreaction was followed in real time and the sensor could be regenerated for repeated use, making it suitable for further clinical development for point-of-care monitoring of insulin. 

Hao et al. [85] reported an approach to a label-free, real-time insulin detection using a graphene aptameric nanosensor. It was based on a graphene field-effect transistor (GFET) and monitored the affinity binding between insulin and its specific aptameric receptor IGA3. The authors suggested that their biosensor could detect insulin with a limit of 35 pmol/L and hence could be developed for clinical use.

A comparison of the detection/signal principle, sample applications, detection limit and linear concentration ranges of different label-free-based biosensors for insulin detection is presented in Table 5.

### 5.4. Other Types of Insulin Biosensors

Further to aptamer- and MIP-based insulin biosensors, there are a few other types reported in the literature, including a sensor based on cyclic voltammetry developed using cobalt hydroxide nanoparticles onto a carbon ceramic electrode [86]. The sensor was used for the detection of insulin in human serum samples and showed high stability, reproducibility and high selectivity, with a limit of detection and sensitivity of 0.11 nmol/L and 11.8 nA/nM, respectively. In 2018, Tan et al. [87] reported a colorimetric assay detecting glucose and insulin simultaneously using gold shell nanorods that possess peroxidase-like activity. In the presence of a peroxidase substrate and glucose oxidase, glucose levels were quantified calorimetrically, and a linear-concentration-dependent relationship was established. An insulin aptamer immobilised on the surface of the nanorods enabled simultaneous insulin detection by masking the catalytic activity of the peroxidase when insulin was bound. The method was developed to measure the glucose/insulin ratio and could potentially be used to differentiate between type I and type II diabetes. An electrochemiluminescent (ECL) biosensor based on carboxyl poly(9,9-dioctyfluorenyl-2,7-diyl) dots (PFO dots) and 3,4,9,10-perylenetetracar-boxylic acid (PTCA) to form an ECL-resonance energy transfer system by labelling two antibodies to detect insulin was published [88]. The biosensor presented good performance with a wide linear range, low detection limit, good stability and selectivity for insulin. Regonda et al. [89] demonstrated the advantages of utilising multiple Si nanochannels (NCs) or nanogratings (NGs) instead of the conventional single nanochannel or nanowire design in Si nanowire field effect transistors for biosensor application for insulin detection in human serum. The NG devices improved performance, reproducibility and device stability and insulin could be detected to a low limit of 10 fmol/L.

## 6. Challenges Associated with Insulin Detection Methods for Point-of-Care Biosensors

A major challenge in insulin detection is to achieve a high enough sensitivity and selectivity in biological fluids. The small size of insulin and its presence at very low levels in biological fluids (Table 1) make its detection more difficult than that of glucose. Insulin levels tend to be lower in biofluids such as sweat, tears, saliva and urine compared to plasma [12,20,21,31,32,90,91,92,93] and point-of-care continuous-monitoring devices should have a sensitive enough detection capability in these settings. Although substantial progress has been made in developing sensitive insulin-detecting biosensors, further optimisation is required to establish the feasibility of each method for use in continuous monitoring mode. In biofluids, detection is hindered by interferents (matrix effect) such as haemoglobin, glucose, ascorbic acid and uric acid as well as drugs like paracetamol, and, therefore, interferents must be carefully assessed for each detection method and for each matrix to ensure selectivity. Knowledge gained from implantable continuous-monitoring glucose biosensors has highlighted problems of signal drifting and sensitivity loss due to the foreign body response and biofouling where a biolayer is formed around the biosensor and restricts its access to the interstitial fluid. Various antifouling approaches such as fabricating coatings with biopolymers have been explored, aiming to maintain accuracy and expand biosensor lifetime [94]. A point-of-care insulin biosensor should be sensitive, selective, as well as stable and ideally non-invasive or minimally invasive for continuous use. Non-invasive biosensors are also less affected by biofouling. Microfluidic devices may also help to remove endogenous interferents and improve selectivity. A point-of-care insulin biosensor should be sensitive, selective as well as reliable and stable and ideally non-invasive or minimally invasive for continuous use. 

Non-invasive biosensors are less affected by biofouling and offer the advantages of being easy to use and pain-free to the user who is in turn more likely to remain compliant. A number of non-invasive insulin and glucose detection techniques using saliva, sweat or tears have been published [20,21,90,91,92]. It is also important to consider whether insulin changes in biofluids such as saliva or sweat reflect accurately and timely what occurs in the circulation. A recent study with a small number of heathy adults showed that levels of insulin in saliva accurately tracked levels found in plasma with a 30-to-45-min delay [95]. Further research is needed to establish the accuracy of each biosensor type and how its measurements reflect true insulin changes in the circulation, taking into account person-to-person variation.

## 7. Conclusions and Future Prospects

While conventional methods for measuring insulin in the clinic are sensitive and robust, point-of-care testing methods are urgently needed to improve the outlook for patients with diabetes. Considerable progress has been made in the development of biosensors with major successes in continuous-glucose-measurement sensors that are integrated into an ‘artificial pancreas’ to deliver the required amount of insulin in diabetic patients. However, the insulin dose is calculated indirectly based on models of the relationship between glucose and insulin and this can lead to dangerous hypoglycaemic and hyperglycaemic episodes. Integration of an insulin biosensor to work in parallel with glucose monitoring will undoubtedly facilitate safe and precise management of diabetes and improve the quality of life of those living with the condition. The detection of insulin is not without challenges though, due to its low concentration in blood (picomolar range) and assay interference with endogenous molecules. Initial attempts to develop insulin biosensors exploited antibody-based interactions. Further biologically inspired biosensor systems in combination with microfluidics are increasingly becoming part of bioelectronic devices designed to measure insulin [51,96,97,98]. 

Aptamer-based and molecularly imprinted biosensors may be considered as improvements on antibody-based biosensors because of their enhanced immobilisation on electrode surfaces, significantly higher stability, high selectivity, high sensitivity and reproducibility. However, their potential interactions with interferants in blood has still not been established. They have however the potential for more economical large-scale production and miniaturisation that are important requirements for point-of-care biosensors. Electrochemical biosensors or those using enhanced chemiluminescence for detection are more sensitive than optical biosensors and this area needs to progress further to develop sensitive enough devices to detect low levels of insulin in biological fluids. Furthermore, latest advances in 3D printing technology and microfluidic systems could contribute to the rapid development of a simultaneous continuous glucose and insulin monitoring for improving diabetes management towards personalised medicine by providing tailored optimal insulin dosages. This system in combination with the continuous and simultaneous monitoring of glucose and insulin as the two key diabetes biomarkers could offer the ultimate solution to diabetes management. Ideally, non-invasive tests detecting insulin in bodily fluids such as saliva are required as well as assay standardisation and compatibility between insulin and glucose monitoring to provide an accurate measurement of glucose and insulin in diabetic individuals. 

The development of accurate and reliable insulin monitoring biosensors relies on drawing from existing and emerging knowledge from different fields. Advancements in the development of biosensors in the glucose and diabetes field have led the way for immense progress in developing biosensors for the detection of other small biomolecules, hormones and biomarkers such as creatinine, cholesterol, cortisol and troponin, and, in turn, progress in these areas will undoubtedly benefit the diabetes field [97,99,100]. The ultimate aim is to develop wearable, sensitive, stable and ideally non-invasive sensor platforms for the concurrent and continuous monitoring of insulin and glucose utilising artificial intelligence techniques to assist with the management of diabetes. 

## Figures and Tables

**Table 1:** Fasting levels of human insulin in different biofluids.

Biofluids	Insulin Normal Levels	References
Serum	30–70 pmol/L	[29]
Plasma	<175 pmol/L	[22,30]
Urine	24–136 pmol/L	[12,31]
Tears	~90 pmol/L	[32]
Saliva	7–28 pmol/L	[20,27,28]

**Table 2:** Sequences of aptamers sensitive to insulin used in biosensor technology.

Aptamer Sequences	References
ILPR: 5′-CAGGGGTGTGGGGACAGGGGTGTGGGG-3′	[63,66]
IGA1: 5′-GGAGGTGGATGGGGAGGGGGAGGTGTGTTT-3′	[9,63]
IGA2: 5′-GGAGGGGGTGGGGAGGGGGCTGGTTGTCC-3′	[63]
IGA3: 5′-GGTGGTGGGGGGGGTGGTAGGGTGTCTTCT-3′	[63,66,67,68,69,70,71]
clGA3: 5′-CCCCACACCCCTGTCCCCACACCCCTG-3′	[69]
IBA2: 5′-CTCTCTCGGTGGTGGGGGGGGTTAGGGTGTCTTCCTCTCTC-3′	[65]

**Table 3:** Comparison of aptamer-based biosensors for detection of insulin.

Transducer	Sample	Detection Limit	Linear Range	References
Fluorescence	Rat serum and human urine	9.97 nmol/L	0–50 nmol/L	[65]
Fluorescence	Human serum	2 nmol/L	2–70 nmol/L	[70]
Fluorescence resonance energy transfer (FRET)	Human plasma	0.6 pmol/L	1 pmol/L–2.0 nmol/L	[71]
Electrochemical	Buffer solution	10 nmol/L	10–200 nmol/L	[66]
Electrochemical	Buffer solution	20 nmol/L	0.02–5 μmol/L	[68]
Flow injection chemiluminescence	Buffer solution	1.6 pmol/L	7.5 pmol/L–5.0 nmol/L	[67]

**Table 4:** Comparison of MIP-based biosensors for detection of insulin.

Transducer	Sample	Detection Limit	Linear Range	References
Dual-electrode QCM	Buffer solution	2.247 nmol/L	2.247–224.7 nmol/L	[73]
QCM chips	Aqueous solution and artificial plasma	18 fmol/L	18 fmol/L–2.247 pmol/L	[74]
Electrochemical	Buffer solution and human plasma	26 fmol/L (buffer) and 81 fmol/L plasma)	50–2000 pmol/L	[75]
Electrochemical	Buffer solution	1.9 pmol/L	20–70 pmol/L	[76]
Electrochemical	Buffer solution	33 fmol/L	0.050–1.40 pmol/L	[77]

**Table 5:** Comparison of label-free-based biosensors for detection of insulin.

Transducer	Sample	Detection Limit	Linear Range	References
IRS	Buffer solution and human islets	4.299 nmol/L	11.235–112.35 nmol/L	[79]
Fluorescence	Buffer solution and human serum	7.07 fmol/L	10 fmol/L–600 pmol/L	[80]
Fluorescence	Buffer solution, human urine and serum	0.1 nmol/L	0.1–1.0 nmol/L	[81]
Electrochemical	Buffer solution and human serum	1.2 pmol/L	5 pmol/L–50 nmol/L	[82]
Electrochemical	Buffer solution	2.26 pmol/L	50–1500 pmol/L	[83]
SPR	Buffer solution and human serum	2.247 pmol/L	2.247–674.1 pmol/L	[84]
Graphene electrical conductance	Buffer solution	35 pmol/L	100 pmol/L–1 μmol/L	[85]
